# Supplementary material for: Physical Inactivity and Sedentary Behaviour among Panamanian Adults: Results from the National Health Survey of Panama (ENSPA) 2019
Source: Int J Environ Res Public Health. 2023 Apr 18;20(8):5554. doi: 10.3390/ijerph20085554 (PMC10138807; doi:10.3390/ijerph20085554)
Supplement: Supplementary file 1 [file ijerph-20-05554-s001.zip › ijerph-2255168-supplementary.pdf]

Manuscript:

Table S1. Selected baseline characteristics of the excluded persons (missing and invalid data) aged 18–69 years by sex. Panama 2019.

Sociodemographic characteristics	All		Men		Women	
	N	Weighted prevalence % (95% CI)	N	Weighted prevalence % (95% CI)	N	Weighted prevalence % (95% CI)
	367,282		181,868	49.5 (44.4–54.6)	185,414	50.5 (45.4–55.6)
Age (years)						
18–29	74,679	20.3 (17.1–24.0)	27,330	15.0 (10.5–21.0)	47,350	25.5 (21.5–30.0)
30–39	98,720	26.9 (22.1–32.2)	49,812	27.4 (19.2–37.4)	48,908	26.4 (22.2–31.0)
40–49	79,073	21.5 (18.8–21.2)	41,867	23.0 (16.5–31.1)	37,206	20.1 (16.2–24.5)
50–59	63,173	17.2 (16.4–18.8)	34,855	19.2 (13.5–31.1)	28,317	15.3 (12.1–19.1)
60–69	51,638	14.1 (14.5–16.7)	28,005	15.4 (10.1–22.8)	23,633	12.8 (9.5–16.8)
Living area						
Urban	280,720	76.4 (73.2–79.3)	131,408	72.2 (66.5–77.4)	149,312	80.5 (77.9–82.9)
Rural	75,540	20.6 (17.7–23.7)	45,074	24.8 (19.8–30.5)	30,466	16.4 (14.1–19.0)
Indigenous	11,023	3.0 (2.5–3.5)	5 387	3.0 (2.3–3.9)	5 636	3.0 (2.6–3.6)
Highest education level achieved						
No formal schooling/special or primary (0–6 years)	70,528	20.0 (16.3–24.3)	40,119	22.5 (16.3–30.3)	30,409	17.4 (13.9–21.5)
Secondary/short-cycle or other (7–13 years)	210,571	59.7 (54.5–64.7)	105,583	59.3 (50.3–67.7)	104,989	60.1 (54.9–61.1)
University (> =13 years)	71,690	20.3 (16.4–24.9)	32,386	18.2 (12.2–26.3)	39,304	22.5 (18.3–27.4)
Ethnic group						
Afro-Panamanian	81,962	23.1 (18.1–29.0)	46,234	25.9 (17.3–36.8)	35,728	20.3 (16.4–25.0)
Multiracial	154,579	43.6 (38.7–48.7)	74,568	41.7 (33.5–50.5)	80,010	45.5 (40.5–50.7)
Indigenous	38,146	10.8 (8.5–13.6)	17,560	9.8 (6.4–14.7)	20,586	11.7 (9.1–15.0)
Caucasian	74,086	20.9 (17.1–25.3)	37,639	21.1 (14.9–28.9)	36,447	20.7 (16.8–25.4)
Asian and others	5 559	1.6 (0.7–3.4)	2 636	1.5 (0.4–5.8)	2 924	1.7 (0.8–3.5)
BMI						
Underweight	7 694	2.3 (1.4–3.8)	2 437	1.5 (0.5–4.3)	5 256	3.1 (1.8–5.3)
Normal	77,889	23.6 (19.6–28.2)	40,741	25.5 (18.6–33.8)	37,149	21.9 (17.8–26.6)
Overweight	120,000	36.4 (31.0–42.2)	70,006	43.8 (34.2–53.9)	49,994	29.5 (24.9–34.4)
Obesity	123,800	37.6 (32.8–42.7)	46,541	29.1 (21.5–38.2)	77,259	45.5 (40.3–50.8)
Self-perception of health status						
Very good or good	319,726	90.3 (87.4–92.5)	166,127	93.0 (88.8–95.7)	153,600	87.5 (83.3–90.7)
Very bad or bad	34,505	9.7 (7.5–12.6)	12,500	7.0 (4.3–11.2)	22,005	12.5 (9.3–16.7)
Smoking						
Yes	38,359	10.8 (8.0–14.5)	27,472	15.4 (10.3–22.3)	10,887	6.2 (4.1–9.4)
No	315,384	89.2 (85.5–92.0)	150,902	84.6 (77.7–89.6)	164,492	93.8 (90.6–95.9
Alcohol consumption						
Yes	119,346	32.5 (27.5–37.9)	78,843	43.4 (34.6–52.6)	40,503	21.8 (17.9–26.4)
No	247,743	67.5 (62.1–72.5)	102,831	56.6 (47.4–65.4)	144,911	78.2 (73.6–82.1)

Source: National Health Survey for Panama (ENSPA) 2019. Percentages are based on weighted data.

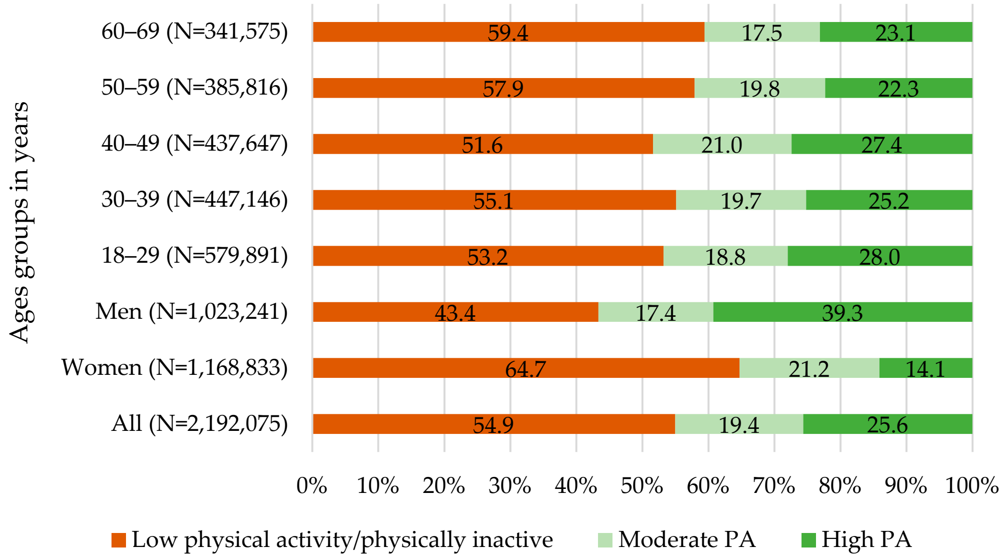

Figure S1. Weighted prevalence of low physical activity category/physically inactive and physical activity (PA) categories stratified by age. Panama, 2019.

Table S2. No physical activity by domain and in any domain and no vigorous-intensity physical activity stratified by age in years old (yo). Panama, 2019.

Domain	All<br>N = 2,192,075<br>Weighted prevalence<br>% (95% CI)	18–29 yo<br>N = 579,891<br>Weighted prevalence<br>% (95% CI)	30–39 yo<br>N = 447,146<br>Weighted prevalence<br>% (95% CI)	40–49 yo<br>N = 437,647<br>Weighted prevalence<br>% (95% CI)	50–59 yo<br>N = 385,816<br>Weighted prevalence<br>% (95% CI)	60–69 yo<br>N = 341,575<br>Weighted prevalence<br>% (95% CI)
Transport	48.7 (47.2–50.2)	47.1 (43.9–50.3)	48.5 (45.3–51.7)	46.7 (43.3–50.2)	53.0 (49.3–56.6)	49.3 (45.4–53.2)
Work / household	80.0 (78.8–81.2)	81.5 (79.1–83.7)	80.4 (77.9–82.7)	76.5 (73.4–79.3)	80.0 (76.8–82.8)	81.6 (78.7–84.2)
Exercise & Sports	82.6 (81.4–83.8)	75.0 (72.2–77.6)	81.3 (78.4–83.9)	84.5 (81.9–86.8)	87.6 (84.8–90.0)	89.2 (86.7–81.2)
In any domain	40.2 (38.7–41.8)	39.0 (35.9–42.3)	39.8 (36.6–43.1)	37.1 (33.9–40.5)	43.2 (39.5–47.0)	43.5 (39.7–47.4)
Intensity						
No Vigorous Intensity PA	77.4 (76.1–78.7)	73.8 (71.0–76.5)	76.6 (73.6–79.3)	76.2 (73.0–79.1)	79.9 (76.5–83.0)	83.6 (80.8–86.1)

Source: National Health Survey for Panama (ENSPA) 2019. CI: Confidence interval.

Table S3. Median and interquartile range (IQR) of minutes spent engaged in sedentary behaviour on average per day by sex among participants aged 18–69 years. Panama, 2019.

Sociodemographic characteristics	Minutes spent in sedentary behaviour on average per day by sex					
	Both		Male		Female	
	N	Median (IQR)	N	Median (IQR)	N	Median (IQR))
	**2,191,889**	**120 (60–240)**	**1,023,143**	**120 (60–240)**	**1,168,745**	**120 (60–240)**
Age (years)						
18–29	579,890	120 (60–240)	244,669	120 (60–240)	335,221	120 (60–240)
30–39	447,101	120 (50–180)	188,236	120 (60–240)	258,864	120 (45–180)
40–49	437,505	120 (45–180)	215,452	120 (60–240)	222,052	120 (60–240)
50–59	385,815	120 (40–180)	192,599	120 (60–180)	193,215	120 (45–240)
60–69	341,575	120 (45–180)	182,184	120 (60–240)	159,390	120 (60–180)
Area						
Urban	1,400,736	120 (60–240)	647,406	120 (60–240)	753,329	120 (60–240)
Rural	661,117	120 (45–180)	314,572	120 (60–240)	346,544	90 (30–180)
Indigenous	130,035	60 (30–120)	61,164	60 (30–120)	68,870	60 (30–120)
Schooling						
No formal schooling/special or primary	551,765	90 (40–180)	257,108	105 (60–180)	294,656	90 (30–180)
Secondary/short-cycle or other	1,235,036	120 (60–240)	595,320	120 (60–240)	639,715	120 (60–240)
University	397,067	180 (60–300)	165,606	180 (60–360)	231,460	180 (60–300)
Body Mass Index (BMI)						
Underweight	44,308	120 (60–240)	22,679	180 (60–240)	21,629	120 (60–300)
Normal	473,459	120 (60–240)	251,182	120 (60–240)	222,277	120 (60–240)
Overweight	688,819	120 (60–240)	339,893	120 (60–240)	348,926	120 (60–180)
Obesity	734,064	120 (60–240)	271,777	120 (60–240)	462,286	120 (60–240)
Self-perception of health status						
Very good or good	1,942,961	120 (60–240)	929,281	120 (60–240)	1,013,680	120 (60–240)
Very bad or bad	247,564	120 (60–300)	93,067	120 (60–300)	154,496	120 (60–300)
Ethnic group						
Caucasian	399,039	120 (60–240)	183,821	120 (60–300)	215,218	120 (60–240)
Afro-Panamanian	335,915	120 (60–240)	172,998	120 (60–240)	162,916	120 (60–240)
Mixed ethnicities	1,133,402	120 (60–240)	528,794	120 (60–240)	604,607	120 (60–240)
Indigenous	257,751	60 (30–180)	110,547	60 (30–120)	147,203	90 (30–180)
Asian and others	64,417	120 (20–180)	26,186	120 (60–180)	38,230	60 (5–180)
Ever tobacco consumption						
No	1,994,773	120 (60–240)	862,460	120 (60–240)	1,132,312	120 (60–240)
Yes	194,915	120 (60–240)	159,887	120 (60–240)	35,027	120 (60–240)
Ever alcohol consumption						
No	1,443,023	120 (60–180)	539,012	120 (60–180)	904,010	120 (60–180)
Yes	748,865	120 (60–240)	484,130	120 (60–240)	264,735	120 (60–300)

Source: National Health Survey for Panama (ENSPA) 2019.

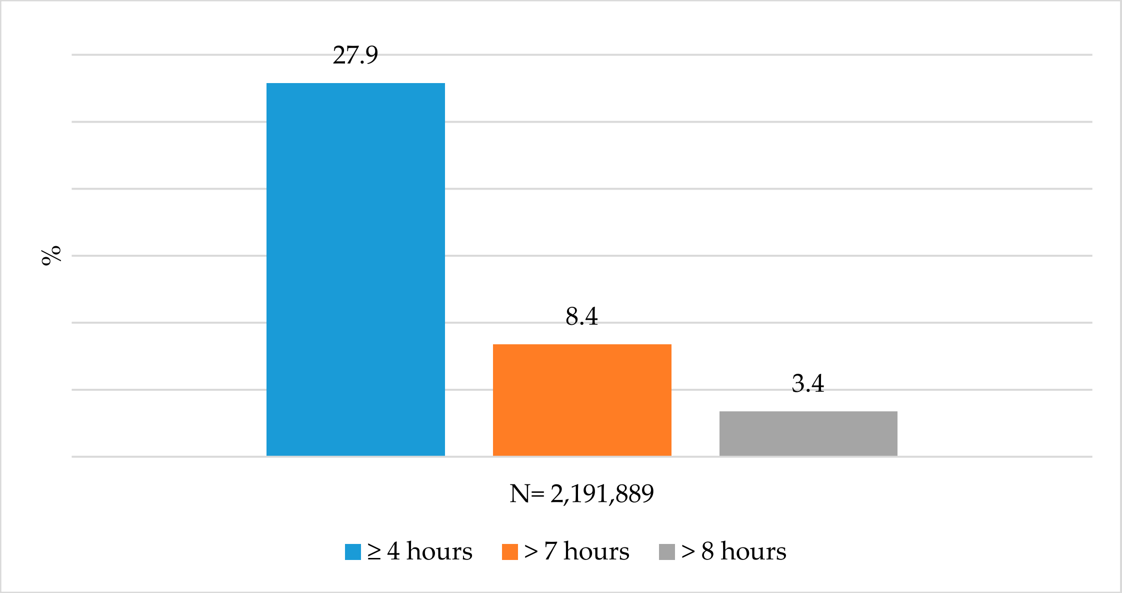

Figure S2. Weighted prevalence of sedentary behaviour according to different cut-off points among participants aged 18–69 years. Panama, 2019.
